# Clinical outcomes of ligamentotaxis in closed phalangeal fractures: a systematic review

**DOI:** 10.1177/17531934251350453

**Published:** 2025-06-19

**Authors:** Joaquin Alfonso Palanca, Michalis Hadjiandreou, Fawz Kazzazi, Honey Ghaffari, Matthew Pywell, Gurjinderpal Singh Pahal

**Affiliations:** 1 Barts and The London School of Medicine and Dentistry, Queen Mary University of London, UK; 2 The Royal London Hand Unit (RLHU), Department of Plastic and Reconstructive Surgery, The Royal London Hospital, UK

**Keywords:** Dynamic external fixation, ligamentotaxis, middle phalanx, phalangeal fractures, proximal interphalangeal joint, proximal phalanx

## Abstract

Closed proximal interphalangeal (PIP) joint and proximal middle phalanx fractures can result in considerable functional disability for the patient. Treatment by ligamentotaxis using a dynamic external fixation (DEF) device appears to be the most promising of several available surgical techniques, although there is no clear consensus. This systematic review of the clinical outcomes of ligamentotaxis in patients with closed phalangeal fractures includes 14 studies and 272 patients. The grand mean QuickDASH (12) and DASH (8) scores show that DEF provides excellent functional results for closed phalangeal fractures at the PIP joint. The incidence of postoperative complications is low and similar to other commonly used surgical techniques, including closed reduction percutaneous pinning, with infection (6.6%) and early osteoarthritis (5.9%) being the most common. Further research in the form of randomized control trials are required to determine the best method of treatment.

## Introduction

The optimal management of closed proximal interphalangeal (PIP) joint and proximal middle phalangeal fractures has long been debated, as none of the methods described has consistently produced better results than another.

Dynamic external fixation (DEF) is a promising surgical technique in the treatment of closed phalangeal fractures. The device uses the principle of ligamentotaxis, applying distal distraction across the PIP joint while providing joint and soft tissue traction to reduce fracture fragments. These devices have provided hand surgeons with an alternative to traditional fixation methods. Dynamic external fixation devices have been shown to provide satisfactory fracture alignment for complex fractures that were previously thought impossible to fix with closed intramedullary Kirschner-wire fixation. The nature of DEF devices allows for early mobilization and rehabilitation while avoiding the inherent risks of soft tissue dissection associated with open surgical procedures. Over the past three decades since dynamic distraction external fixation devices were first described, many different iterations have been produced. This continuous search for improvement makes DEF a promising technique for the treatment of closed phalangeal fractures.

The aim of this systematic review was to present the current evidence on the clinical outcomes of ligamentotaxis in closed phalangeal fractures to better inform clinical practice.

## Methods

This systematic review follows the Preferred Reporting Items for Systematic Reviews and Meta-analysis guidelines (Online Fig. S1) (Page et al., 2021). The review has been prospectively registered in the international prospective register for systematic reviews (PROSPERO) database (CRD42023488566). Study selection criteria were defined with reference to the Population, Intervention, Comparison, Outcome (PICO) Model for clinical questions.

### Population

Patients ≥16 years old with closed phalangeal fractures at the PIP joint level were included. Fractures not at the PIP joint level, open fractures and fractures with associated soft tissue defects were excluded.

### Intervention

Patients undergoing distraction ligamentotaxis using a DEF device such as the Ligamentotaxor® (Arex, Palaiseau, France), Suzuki frame, pins and rubber traction system and Giddins frame. Other methods of distraction ligamentotaxis, including open reduction internal fixation or intra-medullary ligamentotaxis, were excluded.

### Comparator

No control was used in this study.

### Outcome

The primary outcome was the functional status of the patient, measured using the Disabilities of the Arm, Shoulder and Hand (DASH) and QuickDASH (QDASH) scoring systems. The DASH and QuickDASH scores were recorded as a mean score for each paper, with a grand mean calculated for each scoring system. The grand mean is the average of all mean scores.

Secondary outcomes included postoperative complications, need for further procedures, reported pain and stiffness scores, and the affected joint range of motion at final follow-up. Studies that did not clearly report primary outcomes or reported primary outcomes prematurely (defined as less than 1 month postoperatively) were excluded.

### Search strategy

A comprehensive systematic literature search of each database from inception to January 2024 was conducted across five databases: MEDLINE (via PubMed), EMBASE, Web of Science, SCOPUS and Cochrane Reviews databases. A specific search strategy, using a combination of natural language, MeSH terms and Boolean operators was created for each individual database as seen in Appendix S1.

### Study selection

Title and abstract screening was performed independently by the first three authors, followed by full-text review. Any disagreements between the screening authors were resolved by escalation to the senior authors. Articles were selected for inclusion using the pre-defined PICO inclusion and exclusion criteria. Studies were included if they reported functional outcome scores scores using DASH or QDASH in adult patients with closed PIP joint fractures treated with DEF. Studies were excluded if DASH or QDASH were not reported or if they included open fractures or alternative methods of fixation. Studies published in languages other than English were also excluded.

### Data extraction

The following data were extracted and compiled for quantitative analysis:

- Study – main author, year of publication, study title, study design, and inclusion and exclusion criteria of the study.
- Patients – number of patients in the study, sex, mean age, and description and classification of fracture/injury type.
- Intervention – setting of the intervention and description of ligamentotaxis procedure/device
- Follow-up – time between injury and surgery and time between surgery and removal of device (mean duration of fixator), mean follow-up time.
- Clinical outcomes:
- main outcome – DASH and QuickDASH scores;
- secondary outcome – description of complications, reported pain and stiffness scores, additional revision surgery, and PIP joint range of motion of the affected finger.

### Risk of bias assessment

Non-randomized control trials were assessed using the ROBINS-I tool, with papers then being classified as low, moderate, serious or critical risk of bias (Sterne et al., 2016). The quality of included cross-sectional, case control, case reports, case series and cohort studies was assessed using their respective JBI Critical Appraisal Tools (Joanna Briggs Institute, 2021; Munn et al., 2020).

A clinical study was considered to be low risk if it met the following criteria:

- population – consecutive selection of patients, no selection bias, and similar demographics and fracture types;
- intervention – identical device, same team performing the intervention, and same time from injury to intervention; and
- outcome – same follow-up time and rehabilitation protocol for all patients, no deviations from the intended interventions, strict adherence to treatment, no co-interventions, same method of outcome assessment for all patients, and blinding of outcome assessors.

## Results

We identified 8048 studies from five databases. Duplicates (4066) and non-English papers (838) were removed. Title and abstract screening was performed on 3144 studies, of which 3112 were excluded.

**Figure 1.**
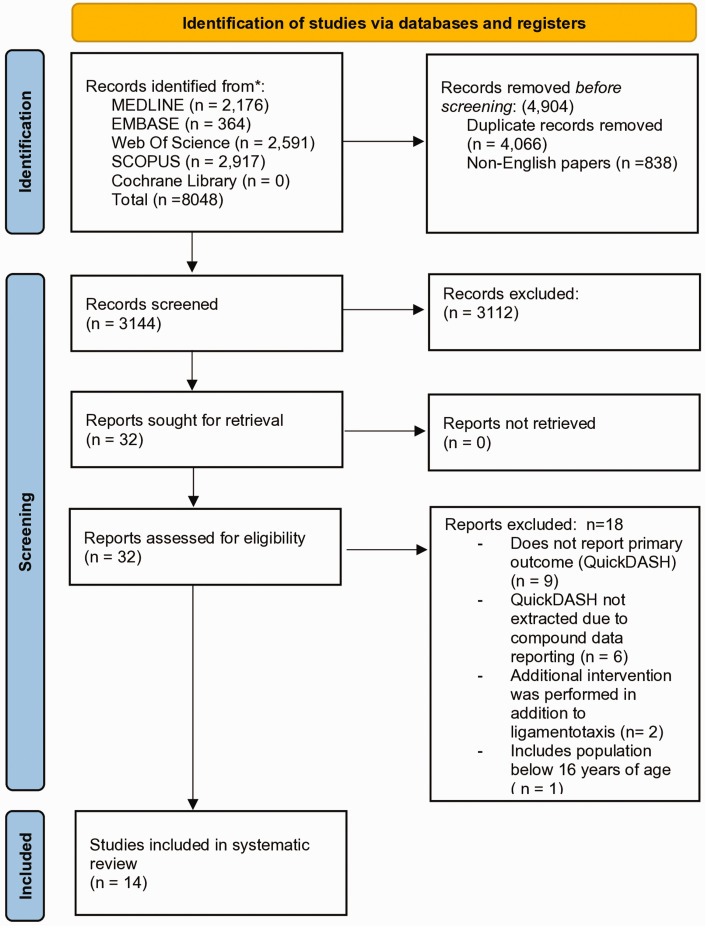
PRISMA flow diagram.

We reviewed the full text of 32 papers; 18 were excluded for the following reasons. Nine papers did not report functional outcome scores. In six papers functional outcome scores could not be extracted owing to reporting of compound data. In two papers co-intervention was performed in addition to ligamentotaxis. One paper included patients under 16 years of age (Figure 1).

Fourteen studies could be included, all with a non-randomized control design. There were eight prospective cohort studies (Colegate-Stone et al., 2015; Damert et al., 2013; Kostoris et al., 2017; Lo et al., 2018; MacFarlane et al., 2015; Pélissier et al., 2015; Sastravaha et al., 2020; Yamamoto et al., 2019), five retrospective cohort series (Abouelela et al., 2020; Awad et al., 2018; Khan and Fahmy, 2006; Mabvuure et al., 2020; Shen et al., 2015) and one prospective case study (Abou Elatta et al., 2016).

### Study characteristics

A total of 272 patients with closed phalangeal fractures treated with DEF devices were included. Twenty-two patients were excluded as they did not meet the inclusion criteria: five patients had open fractures, four patients required open surgery prior to fixation, five had non-PIP joint, proximal or middle phalanx fractures, and eight did not undergo DEF.

The mean age of the included patients was 39 (range 17–89) years, with 74% male and 26% female. The mean interval from injury to surgery was 8.8 (1–43) days. Mean follow-up time was 13.1 (1–119) months. Where the injured finger was reported, 37% involved the little finger, 32% the ring finger, 17% the middle finger, and 14% the index finger.

Fractures that could be classified as volar lip, dorsal lip and pilon fractures showed a relatively even distribution between fracture types with 38% dorsal lip fractures, 33% pilon fractures and 29% volar lip fractures. The detailed classification for each study is shown in Table 1.

**Table 1.** Patient demographic and fracture classification of 272 closed phalangeal fractures treated with dynamic external fixation

Author	No. patients included	No. patients excluded	Sex	Average age (years)*	Finger injured	Fracture classification
Colegate-stone et al., 2015	12		12 male	29 (18–40)	Index – 1Middle – 6Ring – 4Little – 1	Seno classification:Seno 1 (palmar side) – 9Seno 2 (dorsal side) – 2Seno 3 (pilon fracture) – 1
Kostoris et al., 2017	4		3 male:1 female	50 (42–62)	—	Seno classification:Seno 1 (palmar side) – 2Seno 3 (pilon fracture) – 2
Sastravaha et al., 2020	6	73 patients with open fractures4 open surgeries prior to DEF	5 male:1 female	24 (20–31)	Index – 1Middle – 1Ring – 3Little – 1	Dorsal PIPJ fracture dislocation – 3Closed pilon fracture – 2Closed comminuted shaft fracture of middle phalanx – 1
Shen et al., 2015	10		3 male: 7 female	48 (24–79)	Index – 2Middle – 1Ring – 6Little – 1	Schenck grade (6 – IIIA, 4 – IIIB)Palmar lip fracture – 8Pilon Fracture – 2
Abou Elatta et al., 2016	13		8 male: 5 female	37 (26–50)	Index – 1Middle – 0Ring – 4Little – 8	Seno classification:Seno 2 (dorsal lip) – 13
Mabvuure et al., 2020	17	22 patients with open fractures	—	45 (23–67)	Index – 1Middle – 3Ring – 3Little – 10	Seno classification:Seno 1 (palmar side) – 3Seno 2 (dorsal side) – 1Seno 3 (pilon fracture) – 13
Awad et al., 2018	12		8 male:4 female	36 (19–54)	Index – 0Middle – 0Ring – 3Little – 9	—
MacFarlane et al., 2015	28		19 male:9 female	33 (18–67)	Index – 5Middle – 2Ring – 14Little – 7	Seno classification:Seno 1 – 14Seno 2 – 6Seno 3 – 5Seno 5 – 3
Damert et al., 2013	10		7 male:3 female	52 (28–79)	Index – 2Middle – 2Ring – 0Little – 6	AO classification:B1 – 3C1 (Seno I + II) – 5C3 (Seno III + IV + V) – 2
Pélissier et al., 2015	88		69 male:17 female	39 (18–70)	Index – 16Middle – 15Ring – 25Little – 32	Pélissier’s classification:B1 (1), B2 (2), B3 (4), C1 (3), C2 (14), C3 (6), D1 (2), D2 (4), D3 (2), E1 (5), E2 (22), E3 (20), F3 (3)
Abouelela, 2020	33		23 male: 10 female	36 (18–70)	—	Pélissier’s classification:Type B (simple articular fracture) – 33%Type C (pilon fracture) – 21%Type E (Palmar lip fracture) – 33%
Khan and Fahmy, 2006	15	55 fractures not of PIPJ	14 male: 1 female	36 (18–51)	Index – 3Middle – 4Ring – 6Little – 2	—
Lo et al., 2018	20		15 male: 5 female	38 (21–62)	Index – 1Middle – 4Ring – 5Little – 10	Kiefhaber and Sterne’s classification:Pilon fracture – 9Palmar lip fracture (unstable) – 6Dorsal lip fracture (unstable) – 4Palmar lip fracture (tenuous) – 1
Yamamoto et al., 2019	4	88 did not undergo DEF	—	46 (36–53)	Index – 1Middle – 1Ring – 1Little – 1	—

* Values are given as mean (range). —, Value not reported. No., Number; DEF, dynamic external fixation; PIPJ, proximal interphalangeal joint.

External fixation devices varied between the studies. Eight studies used the Ligamentotaxor® device, two used a modified pins and rubber traction system and one used the S-Quattro device. One paper each reported use of a syringe external fixator, a cerclage wire dynamic external fixator and the Ichi fixator system.

Tables 1 and 2 list patient demographics and study characteristics, respectively.

**Table 2.** Summary of dynamic external fixation device treatment protocols

Author	Dynamic External Fixation device	Timbe between injury to surgery (days)*	Mean duration of fixator device (weeks)	Mean follow up time (months)*
Colegate-stone et al., 2015	Ligamentotaxor® device	10 (2–20)	6	4 (1–6)
Kostoris et al., 2017	Ligamentotaxor® device	7 (3–9)	6 (3–9)	6 (2–11)
Sastravaha et al., 2020	Syringe External Fixator	2 (1–5)	4–6	6
Shen et al., 2015	Dynamic Distraction External Fixator (rubber bands) PRTS	28 (21–43)	4	24 (10–36)
Abou Elatta et al., 2016	Dynamic External Fixator (cerculage wire)	7 (3–13)	5	18 (6–92)
Mabvuure et al., 2020	Ligamentotaxor® device	12 (2–35)	6	6 (8–18)
Awad et al., 2018	Ligamentotaxor® device	4 (2–7)	6	6 (3–22)
MacFarlane et al., 2015	Ligamentotaxor® device	7 (1–18)	4–6	22 (6–52)
Damert et al., 2013	Ligamentotaxor® device	4	7	15
Pélissier et al., 2015	Ligamentotaxor® device	–	5	15 (6–38)
Abouelela, 2020	Ligamentotaxor® device	9 (1–10)	5	28 (12–108)
Khan and Fahmy, 2006	S – Quattro device	6 (1–16)	4–6	13 (7–18)
Lo et al., 2018	Modified pins and Rubber Traction System (Deshmukh)	10 (1–28)	6	8 (1–27)
Yamamoto et al., 2019	Ichi fixator system	—	—	—

* Values are given as mean (range). — Value not reported. PRTS: Pins and rubber traction system.

### Clinical and functional outcomes

**Figure 2.**
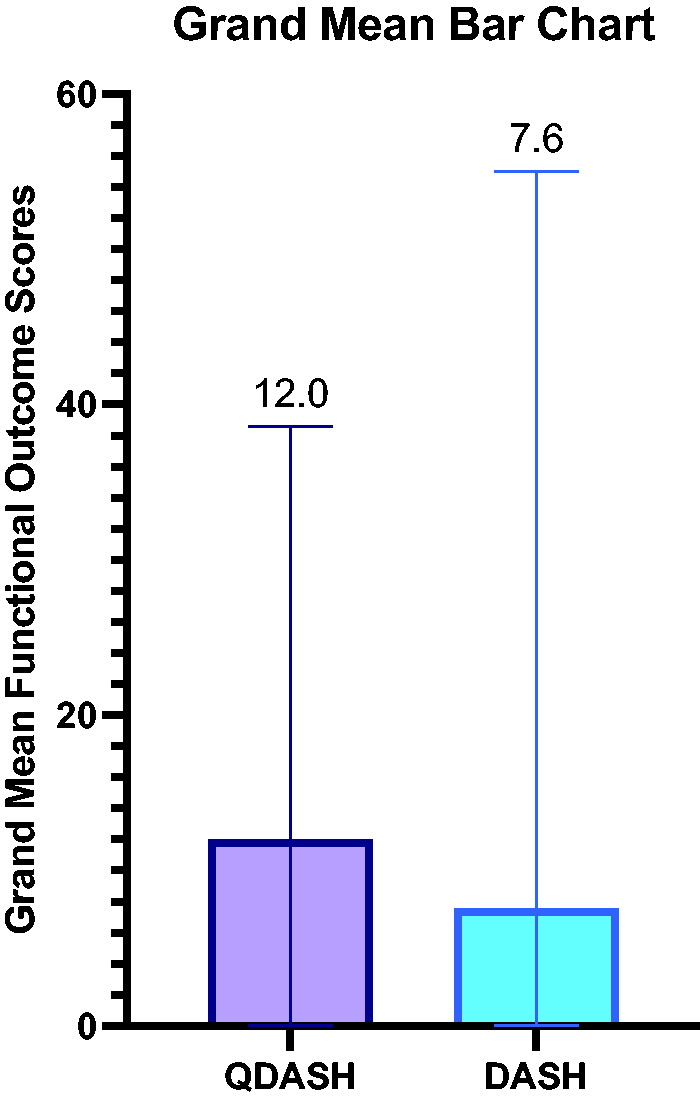
Grand mean functional outcome scores.

**Figure 3.**
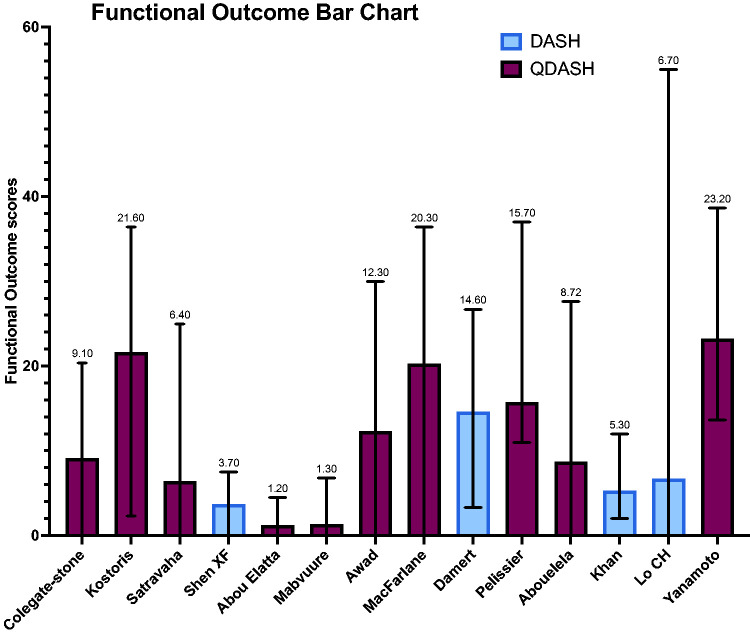
Mean DASH scores all included papers.

For the primary outcomes, 10 studies reported functional outcomes using the QDASH score, with a grand mean of 12 (0–39). Four studies reported functional outcomes using the DASH score, with a grand mean of 8 (0–55) (Figures 2 and 3). Raw functional outcome scores were only reported in seven of 14 studies (Khan and Fahmy, 2006; Kostoris et al., 2017; Mabvuure et al., 2020; Pélissier et al., 2015; Sastravaha et al., 2020; Shen et al., 2015; Yamamoto et al., 2019).

**Figure 4.**
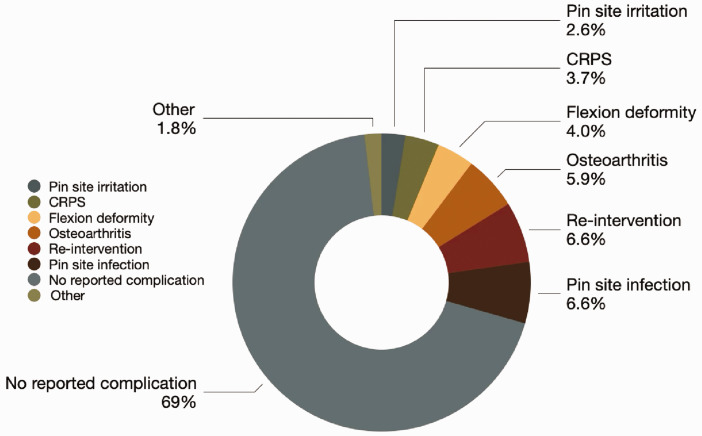
Secondary outcome complication rates.

Regarding secondary outcomes, most patients treated with DEF (69%, *n* = 187), reported no complications. The most common complications after surgery were re-intervention (6.6%, *n = *18), pin-site infection (6.6%, *n* = 18) and osteoarthritis (5.9%, *n* = 16) (Figure 4, Online Fig. S2). Infections that could not be treated with oral antibiotics were the most common reason for re-intervention at 44% (*n* = 8) (Online Fig. S3). The mean active PIP joint ROM was 77°, with six studies reporting ROM less than 75° (Online Fig. S4) (Abouelela et al., 2020; Colegate-Stone et al., 2015; Damert et al., 2013; Kostoris et al., 2017; Lo et al., 2018; Mabvuure et al., 2020). Other complications included flexion deformities (4.0%, *n = *11), complex regional pain syndrome (3.7%, *n = *10) and pin-site irritation (2.6%, *n = *7). A summary of all secondary outcomes is shown in Table 3.

**Table 3:** Summary of functional outcome scores and complications of closed phalangeal fractures treated with dynamic external fixation devices

Author	Mean QuickDASH*	Mean DASH*	Complications	Re-intervention	Active mean PIPJ ROM (°)	Outcome scores*
Colegate-stone et al., 2015	9.1 (0–20.4)		Pin-site infection – 2	Removal of wire (infection) – 1	63	—
Kostoris et al., 2017	21.6 (2.3–36.4)		Minimal rotational defect – 1		61	VAS score – 2.2
Sastravaha et al., 2020	6.4 (0–25)				89	NRS pain score – 1.3
Shen et al., 2015		3.7 (0–7.5)			84	MHQ – 97.3
Abou Elatta et al., 2016	1.2 (0–4.5)		Hyperextension of DIPJ and extension lag of PIPJ – 4		90	—
Mabvuure et al., 2020	1.3 (0–6.8)		Pin-site infection – 1Pin-site irritation – 3Complex Regional Pain Syndrome (CRPS) – 2Severe OA– 1Osteomyelitis – 1	Removal of wire (infection) – 1Arthroplasty of PIPJ (OA) – 1Removal of wire (osteomyelitis) – 1	71	—
Awad et al., 2018	12.3 (0–30)		Pin-site infection – 4Fixed flexion deformity – 5		—	VAS score – 1.5Grip strength – 78%
MacFarlane et al., 2015	20.3 (0–36.4)		Pin-site infection – 2Pin-site irritation – 4Radiographic loss of joint space (OA) – 12DIPJ extensor lag – 2Loss of reduction – 1	PIPJ replacement (OA) – 1Removal of wire (infection) – 1Removal of fixator (irritation) – 4Replacement of fixator (loss of reduction) – 1	85	—
Damert et al., 2013		14.6 (3.3–26.7)	Trauma to external fixator – 1	Additional K-wire osteosynthesis (trauma to fixator) – 1	73	Grip strength – 71%
Pélissier et al., 2015	15.7 (11–37)		Pin-site infection – 4Osteoarthritis – 1Complex Regional Pain Syndrome – 4	Removal of fixator (osteoarthritis) – 1	81	Pain on exertion – 47%Constant pain – 26%
Abouelela, 2020	8.7 (0–27.6)		Pin–site infection – 1Cold intolerance and persistent swelling (CRPS) – 4Radiological signs of OA – 2		66	Pain on exertion – 35%
Khan and Fahmy, 2006		5.3 (2–12)	Trauma to external fixator – 1	Re-application of fixator (trauma) – 1	95	VAS pain score – 2
Lo et al., 2018		6.7 (0–55)	Pin-site infection – 4	Removal of fixator (infection) – 4	62	NRS pain– 0Pain on exertion – 2
Yamamoto et al., 2019	23.2 (13.6–38.6)		—	—	—	VAS pain score – 1.15

* Values are given as mean (range). —, Value not reported. DIPJ, Distal interphalangeal joint; PIPJ, proximal interphalangeal joint; OA, osteoarthritis; ROM, range of motion; VAS, pain visual analogue score; NRS, numerical rating scale; MHQ, Michigan Hand Outcomes Questionnaire.

### Risk assessment

**Figure 5.**
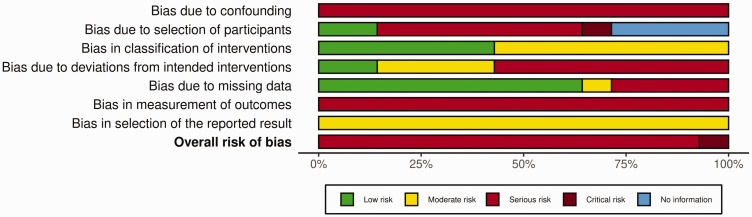
ROBINS-I risk of bias summary plot.

All 14 papers were assessed for risk of bias using the JBI Critical Appraisal Checklist (Joanna Briggs Institute, 2021; Munn et al., 2020). Risk of bias was further assessed using the ROBINS-I tool (Sterne et al., 2016). The overall risk of bias was critical in one paper (Awad et al., 2018) and serious in 13 papers (Abou Elatta et al., 2016; Abouelela et al., 2020; Colegate-Stone et al., 2015; Damert et al., 2013; Khan and Fahmy, 2006; Kostoris et al., 2017; Lo et al., 2018; Mabvuure et al., 2020; MacFarlane et al., 2015; Pélissier et al., 2015; Sastravaha et al., 2020; Shen et al., 2015; Yamamoto et al., 2019). No information was available for the assessment of ‘bias in participant selection’ for four papers, which was due to a lack of clear inclusion and exclusion criteria. A summary of the risk of bias analysis is shown in Figure 5 and Online Fig. S5.

## Discussion

This systematic review confirms that DEF ligamentotaxis is an excellent alternative to traditional methods of fixation for closed phalangeal fractures at the PIP joint level. The grand mean QDASH (12) and DASH (8) scores highlight a near normal functional outcome after the procedure.

The incidence of postoperative complications was low with 69% of patients reporting no adverse events. This review also identified postoperative infection (6.6%) and early osteoarthritis (5.9%) as the two most common sequelae of DEF ligamentotaxis.

Osteoarthritis is an often overlooked complication, and the numbers are likely to be under-reported. In this paper, five studies had a mean follow-up of less than 7 months with many more patients lost to follow-up even earlier (Awad et al., 2018; Colegate-Stone et al., 2015; Kostoris et al., 2017; Mabvuure et al., 2020; Sastravaha et al., 2020). This short follow-up is insufficient to observe the development of osteoarthritis.

Our findings have important implications for clinical practice. Compliance to the intensive postoperative hand therapy should be assessed prior to surgery, and a fully integrated multidisciplinary team is critical to ensure timely detection of these complications.

Our results are comparable with those of two other systematic reviews. Gianakos et al. (2020) included 16 studies and 224 patients. They reported higher DASH (9 vs. 8) and QuickDASH (19 vs. 12) scores than our review. Secondary outcomes were largely consistent with similar ROM (80 vs. 77°), lower incidence of re-intervention (4 vs. 7%) and osteoarthritis (4.9 vs. 5.9%); however, reported incidences of infection were more than double our findings (16 vs. 7%) (Gianakos et al., 2020). Demino et al. (2021) included 25 studies and 427 patients and did not report functional outcomes; however, the average ROM at final follow-up (81 vs. 77°) was marginally higher than our study. A direct comparison is difficult owing to the inclusion of open fractures in both papers.

Several alternative surgical techniques are commonly used in the management of PIP joint fractures, including closed reduction and percutaneous pinning (CRPP). Comparative studies have demonstrated similar function outcomes between CRPP and DEF. Gaio and Kruse (2025) and Gianakos et al. (2020) reported mean DASH scores of 8 and 8 respectively, closely aligning with the grand mean DASH score of 8 in this systematic review. Randomized control trials by El-Saeed et al. (2019) and Zhang et al. (2019) found even lower QDASH scores for CRPP (6 and 6 vs. 12). Additionally, reported incidences of infection (2.8, 7.3 and 3.7%) and re-intervention (5.0, 11, 0 and 0%) are generally lower than those associated with DEF (El-Saeed et al., 2019; Gaio and Kruse, 2025; Gianakos et al., 2020; Zhang et al., 2019).

Caution should be taken when making direct comparisons between these two surgical techniques. The treatment a patient receives is generally determined by the severity of the fracture. Closed reduction and percutaneous pinning is typically used for simple PIP joint fractures with less articular involvement while DEF is indicated for more severe fracture patterns (El-Saeed et al., 2019; Gianakos et al., 2020). It is therefore difficult to determine whether the difference in functional outcome and complications seen is related to the surgical technique or to the severity of the fractures treated.

Use of the DEF device has limitations. In a subset of intra-articular fractures, where the bone fragments lack ligamentous attachment, DEF may be less effective. In these cases, a combined approach with open surgery is therefore recommended to elevate and align the central fragment and achieve optimal joint congruency (Demino et al., 2021).

This systematic review has several limitations. All papers were considered to have a serious or critical risk of bias in more than one ROBINS-I domain. The heterogeneity of fracture patterns within study cohorts introduced important confounding biases. The variability in postoperative rehabilitation and length of follow-up protocols made comparison between studies extremely difficult. In additon, limited reporting of raw functional outcome scores and incomplete datasets precluded a robust meta-analysis of the results. The lack of standardization of reported outcomes resulted in functional outcomes being measured using two different scores, DASH and QDASH. Direct comparison between these is inappropriate, further limiting the analysis. Finally, the exclusion of over 800 non-English papers probably omitted relevant publications that met our PICO inclusion criteria. This introduced selection bias and limited the scope of our systematic review.

Given the limitations of this and other systematic reviews, there is a need for further research in the form of randomized control trials to determine the best form of fixation in closed phalangeal PIP joint fractures.

## Supplemental Material

sj-pdf-1-jhs-10.1177_17531934251350453 - Supplemental material for Clinical outcomes of ligamentotaxis in closed phalangeal fractures: a systematic review Supplemental material, sj-pdf-1-jhs-10.1177_17531934251350453 for Clinical outcomes of ligamentotaxis in closed phalangeal fractures: a systematic review by Joaquin Alfonso Palanca, Michalis Hadjiandreou, Fawz Kazzazi, Honey Ghaffari, Matthew Pywell and Gurjinderpal Singh Pahal in Journal of Hand Surgery (European Volume)

sj-pdf-2-jhs-10.1177_17531934251350453 - Supplemental material for Clinical outcomes of ligamentotaxis in closed phalangeal fractures: a systematic review Supplemental material, sj-pdf-2-jhs-10.1177_17531934251350453 for Clinical outcomes of ligamentotaxis in closed phalangeal fractures: a systematic review by Joaquin Alfonso Palanca, Michalis Hadjiandreou, Fawz Kazzazi, Honey Ghaffari, Matthew Pywell and Gurjinderpal Singh Pahal in Journal of Hand Surgery (European Volume)

sj-pdf-3-jhs-10.1177_17531934251350453 - Supplemental material for Clinical outcomes of ligamentotaxis in closed phalangeal fractures: a systematic review Supplemental material, sj-pdf-3-jhs-10.1177_17531934251350453 for Clinical outcomes of ligamentotaxis in closed phalangeal fractures: a systematic review by Joaquin Alfonso Palanca, Michalis Hadjiandreou, Fawz Kazzazi, Honey Ghaffari, Matthew Pywell and Gurjinderpal Singh Pahal in Journal of Hand Surgery (European Volume)

sj-pdf-4-jhs-10.1177_17531934251350453 - Supplemental material for Clinical outcomes of ligamentotaxis in closed phalangeal fractures: a systematic review Supplemental material, sj-pdf-4-jhs-10.1177_17531934251350453 for Clinical outcomes of ligamentotaxis in closed phalangeal fractures: a systematic review by Joaquin Alfonso Palanca, Michalis Hadjiandreou, Fawz Kazzazi, Honey Ghaffari, Matthew Pywell and Gurjinderpal Singh Pahal in Journal of Hand Surgery (European Volume)

sj-pdf-5-jhs-10.1177_17531934251350453 - Supplemental material for Clinical outcomes of ligamentotaxis in closed phalangeal fractures: a systematic review Supplemental material, sj-pdf-5-jhs-10.1177_17531934251350453 for Clinical outcomes of ligamentotaxis in closed phalangeal fractures: a systematic review by Joaquin Alfonso Palanca, Michalis Hadjiandreou, Fawz Kazzazi, Honey Ghaffari, Matthew Pywell and Gurjinderpal Singh Pahal in Journal of Hand Surgery (European Volume)

sj-pdf-6-jhs-10.1177_17531934251350453 - Supplemental material for Clinical outcomes of ligamentotaxis in closed phalangeal fractures: a systematic review Supplemental material, sj-pdf-6-jhs-10.1177_17531934251350453 for Clinical outcomes of ligamentotaxis in closed phalangeal fractures: a systematic review by Joaquin Alfonso Palanca, Michalis Hadjiandreou, Fawz Kazzazi, Honey Ghaffari, Matthew Pywell and Gurjinderpal Singh Pahal in Journal of Hand Surgery (European Volume)
