# Supplementary material for: Clinical outcomes of ligamentotaxis in closed phalangeal fractures: a systematic review
Source: J Hand Surg Eur Vol. 2025 Jun 19;51(1):14–24. doi: 10.1177/17531934251350453 (PMC12705889; doi:10.1177/17531934251350453)
Supplement: sj-pdf-1-jhs-10.1177_17531934251350453 - Supplemental material for Clinical outcomes of ligamentotaxis in closed phalangeal fractures: a systematic review [file sj-pdf-1-jhs-10.1177_17531934251350453.pdf]

Search strategy MEDLINE (Via PubMed)

((Phalanx [tiab] OR Phalange* [tiab] OR Finger* [tiab] OR Finger bone [tiab] OR Interphalangeal joint* [tiab] OR Proximal Interphalangeal Joint [tiab] OR PIPJ [tiab] OR Finger Interphalangeal Joint [tiab] OR Proximal phalanx [tiab] OR Proximal phalan* [tiab] OR Middle phalanx [tiab] OR Middle phalange* [tiab] OR Finger Phalanges [MeSH] OR Finger Joint [MeSH] OR Fingers [MeSH]) AND (Fractur* [tiab] OR Complex fractur* [tiab] OR Fracture dislocation [tiab] OR Fracture-dislocation [tiab] OR Intra-articular fractur* [tiab] OR Pilon fractur* [tiab] OR Fracture subluxations [tiab]OR Closed fractur* [tiab] OR Fractures, Bone [MeSH] OR Intra-articular fractures [MeSH] OR Fractures, Closed [MeSH] OR Fracture Dislocation [MeSH])) AND (Ligamentotaxis [tiab] OR Ligamentous traction [tiab] OR Ligamentous Reduction [tiab] OR Ligamentotaxor [tiab] OR Distraction [tiab] OR Distraction Ligamentotaxis [tiab] OR Dynamic distraction [tiab] OR Dynamic traction [tiab] OR Dynamic fixator [tiab] OR Dynamic external fixat* [tiab] OR Dynamic digital traction [tiab] OR Dynamic external traction [tiab] OR External fixat* [tiab] OR Skeletal traction [tiab] OR Closed reduction [tiab] OR Pins and rubber traction system [tiab] OR Giddins frame [tiab] OR Suzuki Frame [tiab] OR Banjo Frame [tiab] OR Kirschner□wire [tiab] OR K-wire [tiab] OR External fixators [MeSH] OR Fracture fixation [MeSH] OR Closed Fracture Reduction [MeSH])

Search strategy EMBASE

('finger'/de OR 'finger phalanx'/de OR 'proximal phalanx'/de OR 'middle phalanx'/de OR 'proximal interphalangeal joint'/de OR 'phalanx'/de OR 'phalange*':ab,ti OR 'finger bone':ab,ti OR 'interphalangeal joint*':ab,ti OR 'pipj':ab,ti OR 'finger interphalangeal joint':ab,ti OR 'proximal phalanx*':ab,ti OR 'middle pahalnge*':ab,ti) AND ('fracture'/de OR 'fracture dislocation'/de OR 'intraarticular fracture'/de OR 'phalanx fracture'/de OR 'pilon fracture'/de OR 'complex fractur*':ab,ti OR 'fracture-dislocation':ab,ti OR 'fracture subluxation':ab,ti OR 'closed fractur*':ab,ti) AND ('ligamentotaxis'/de OR 'closed fracture reduction'/de OR 'fracture external fixation'/de OR 'ligamentous traction':ab,ti OR 'ligamentous reduction':ab,ti OR 'ligamentotaxor':ab,ti OR 'distraction ligamentotaxis':ab,ti OR 'dynamic distraction':ab,ti OR 'dynamic traction':ab,ti OR 'dynamic fixator':ab,ti OR 'dynamic external fixat*':ab,ti OR 'dynamic digital traction':ab,ti OR 'dynamic external traction':ab,ti OR 'external fixat*':ab,ti OR 'skeletal traction':ab,ti OR 'pins and rubber traction system':ab,ti OR 'giddins frame':ab,ti OR 'suzuki frame':ab,ti OR 'banjo frame':ab,ti OR 'kirschner-wire':ab,ti OR 'k-wire':ab,ti OR 'fracture fixation':ab,ti)

Search strategy web of science

TS=(Phalanx OR Phalange* OR Finger* OR "Finger bone" OR "Interphalangeal joint*" OR "Proximal Interphalangeal Joint" OR PIPJ OR "Finger Interphalangeal Joint" OR "Proximal phalanx" OR "Proximal phalanx*" OR "Middle phalanx" OR "Middle phalange*" OR "Finger phalanges" OR "Finger Joint") AND TS=(Fracture* OR "Complex fractur*" OR "Fracture dislocation" OR "Fracture-dislocation" OR "Intra-articular fractur*" OR "Pilon fractur*" OR "Fracture subluxations" OR "Closed fractur*") AND TS=(ligamentotaxis OR "Ligamentous traction" OR "Ligamentous reduction" OR Ligamentotaxor OR "Distraction Ligamentotaxis" OR "Dynamic distraction" OR "Dynamic traction" OR "Dynamic fixator" OR "Dynamic external fixat*" OR "Dynamic digital traction" OR "Dynamic external traction" OR "External fixat*" OR "Skeletal traction" OR "Closed reduction" OR "Pins and rubber traction system" OR "Giddins frame" OR "Suzuki Frame" OR "Banjo Frame" OR "Kirschner-wire" OR "K-wire" OR "Fracture fixation" OR "Closed Fracture Reduction")

Search strategy SCOPUS

TITLE-ABS-KEY (phalanx OR phalange* OR finger* OR "Finger bone" OR "Interphalangeal joint*" OR "Proximal Interphalangeal Joint" OR pipj OR "Finger Interphalangeal Joint" OR "Proximal phalanx" OR "Proximal phalanx*" OR "Middle phalanx" OR "Middle phalange*" OR "Finger phalanges" OR "Finger Joint") AND TITLE-ABS-KEY-AUTH (fracture* OR "Complex fractur*" OR "Fracture dislocation" OR "Fracture-dislocation" OR "Intra-articular fractur*" OR "Pilon fractur*" OR "Fracture subluxations" OR "Closed fractur*") AND TITLE-ABS-KEY (ligamentotaxis OR "Ligamentous traction" OR "Ligamentous reduction" OR ligamentotaxor OR "Distraction Ligamentotaxis" OR "Dynamic distraction" OR "Dynamic traction" OR "Dynamic fixator" OR "Dynamic external fixat*" OR "Dynamic digital traction" OR "Dynamic external traction" OR "External fixat*" OR "Skeletal traction" OR "Closed reduction" OR "Pins and rubber traction system" OR "Giddins frame" OR "Suzuki Frame" OR "Banjo Frame" OR "Kirschner-wire" OR "K-wire" OR "Fracture fixation" OR "Closed Fracture Reduction")

Search strategy Cochrane Review database

(fractur*:ti,ab,kw AND phalange*:ti,ab,kw)
