# Supplementary figures and images for: Clinical outcomes of ligamentotaxis in closed phalangeal fractures: a systematic review
Source: J Hand Surg Eur Vol. 2025 Jun 19;51(1):14–24. doi: 10.1177/17531934251350453 (PMC12705889; doi:10.1177/17531934251350453)

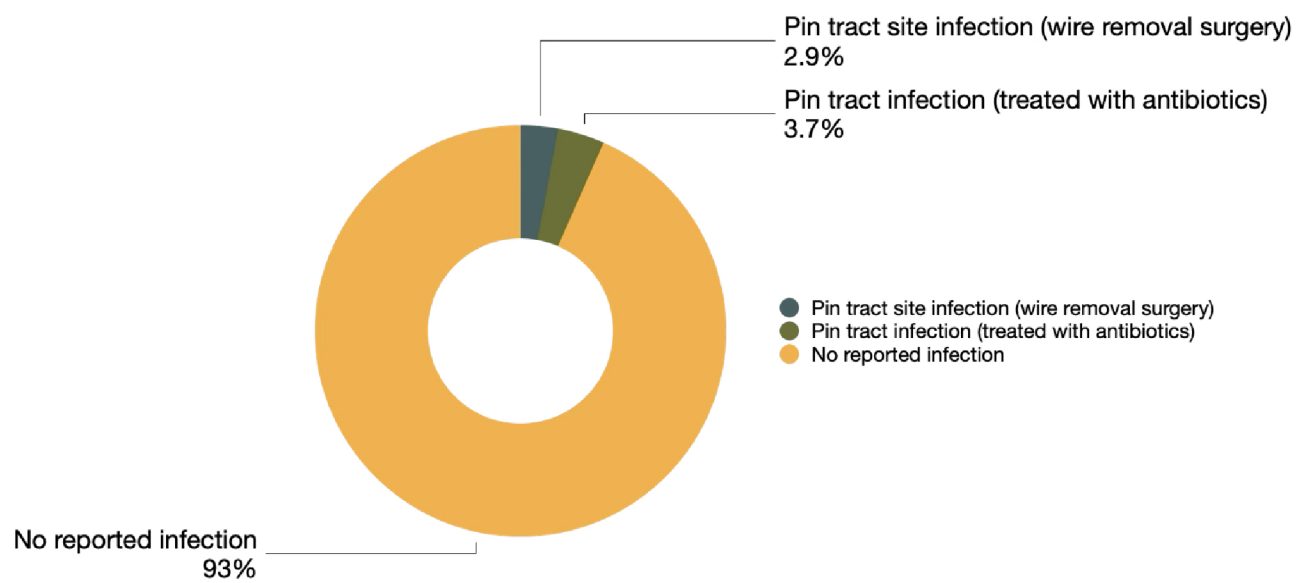

Supplement: sj-pdf-3-jhs-10.1177_17531934251350453 - Supplemental material for Clinical outcomes of ligamentotaxis in closed phalangeal fractures: a systematic review [file sj-pdf-3-jhs-10.1177_17531934251350453.pdf]

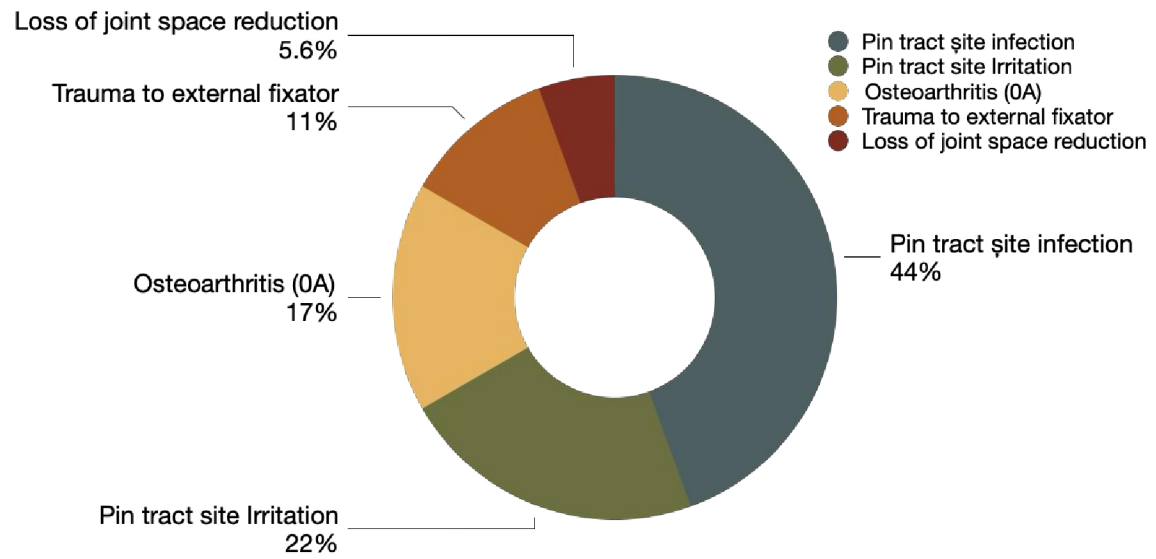

Supplement: sj-pdf-4-jhs-10.1177_17531934251350453 - Supplemental material for Clinical outcomes of ligamentotaxis in closed phalangeal fractures: a systematic review [file sj-pdf-4-jhs-10.1177_17531934251350453.pdf]

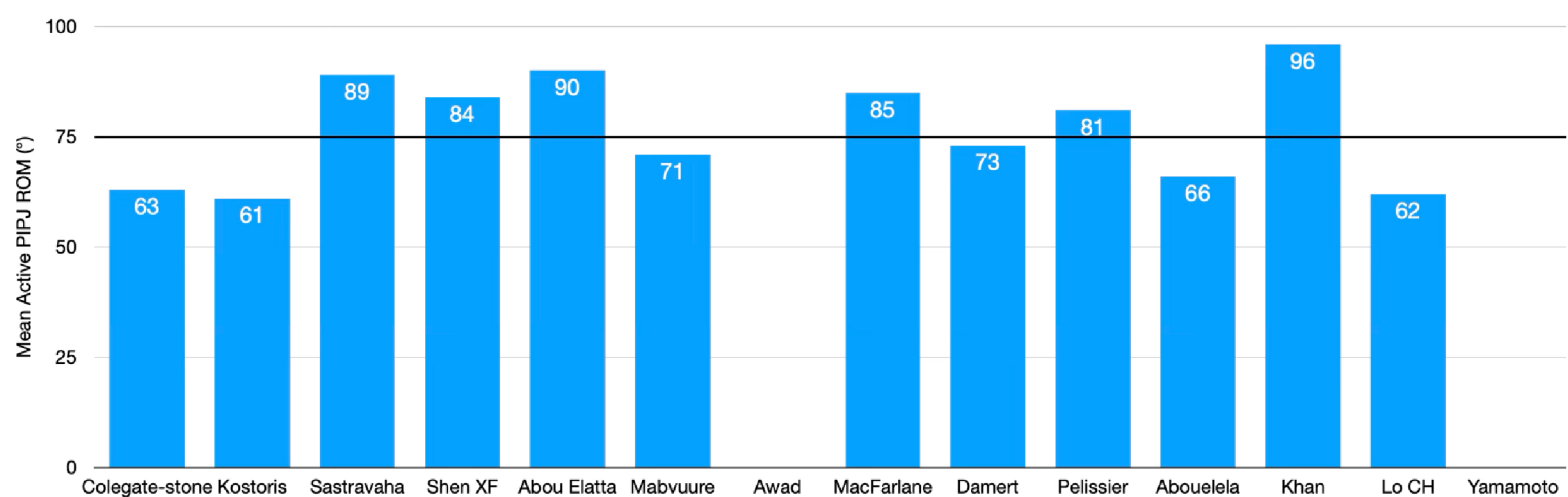

Supplement: sj-pdf-5-jhs-10.1177_17531934251350453 - Supplemental material for Clinical outcomes of ligamentotaxis in closed phalangeal fractures: a systematic review [file sj-pdf-5-jhs-10.1177_17531934251350453.pdf]
