# Supplementary material for: Clinical outcomes of ligamentotaxis in closed phalangeal fractures: a systematic review
Source: J Hand Surg Eur Vol. 2025 Jun 19;51(1):14–24. doi: 10.1177/17531934251350453 (PMC12705889; doi:10.1177/17531934251350453)
Supplement: sj-pdf-6-jhs-10.1177_17531934251350453 - Supplemental material for Clinical outcomes of ligamentotaxis in closed phalangeal fractures: a systematic review [file sj-pdf-6-jhs-10.1177_17531934251350453.pdf]

Risk of bias domains

Study	D1	D2	D3	D4	D5	D6	D7	Overall
Colegate-Stone, 2015	X	X	+	X	+	X	-	X
Kostoris, 2017	X	?	+	+	+	X	-	X
Sastravaha, 2020	X	X	-	X	+	X	-	X
Shen XF, 2015	X	X	+	-	+	X	-	X
Abou Elatta, 2016	X	X	+	X	+	X	-	X
Mabvuure, 2020	X	?	-	X	X	X	-	X
Awad, 2018	X	!	-	X	X	X	-	!
MacFarlane, 2015	X	+	+	X	X	X	-	X
Damert, 2013	X	+	+	-	+	X	-	X
Pelissier, 2015	X	X	-	-	-	X	-	X
Abouelela, 2020	X	X	-	+	+	X	-	X
Khan, 2006	X	?	-	-	+	X	-	X
Lo CH, 2018	X	X	-	X	X	X	-	X
Yanamoto, 2019	X	?	-	X	+	X	-	X

Domains:
D1: Bias due to confounding.
D2: Bias due to selection of participants.
D3: Bias in classification of interventions.
D4: Bias due to deviations from intended interventions.
D5: Bias due to missing data.
D6: Bias in measurement of outcomes.
D7: Bias in selection of the reported result.

Judgement
! Critical
X Serious
- Moderate
+ Low
? No information
